# Traditional Chinese medicine for cardiovascular disease: efficacy and safety

**DOI:** 10.3389/fcvm.2024.1419169

**Published:** 2024-12-03

**Authors:** Youwei Lin, Yuanshan Han, Yuhong Wang

**Affiliations:** ^1^ Institute of Innovation and Applied Research in Chinese Medicine, Hunan University of Chinese Medicine, Changsha, China; ^2^ Scientific Research Department, The First Hospital of Hunan University of Chinese Medicine, Changsha, China

**Keywords:** cardiovascular diseases, Chinese patent medicine, hypertension, cardiovascular events, death events

## Abstract

In China and other Asian nations, traditional medicine has long been utilized in the treatment of cardiovascular diseases (CVD). While Chinese authorities have incorporated traditional Chinese medicine (TCM) treatment experiences as a supplementary guide for CVD, its international recognition remains limited due to a scarcity of high-quality and reliable randomized controlled trials (RCTs) evidence. The purpose of this study was to examine the clinical outcomes with TCM for CVD after the recent publication of large trials adding >20,000 individuals to the published data. Here, we systematically reviewed 55 published RCTs (modified Jadad scores > 4) in the past 20 years, involving a total of 36,261 patients. In most studies, TCM has been associated with significant improvements in alternative endpoints such as hypertension, coronary heart disease, stroke and heart failure. A total of 19 trials reported on primary outcomes such as cardiovascular events and death events. During the follow-up period, some Chinese patent medicines can effectively reduce the “hard” endpoints of coronary heart disease, stroke, and heart failure, the overall trend of cardiovascular outcomes is lower. The risk of adverse effects was not significantly increased compared to the control group, suggesting its potential as an alternative approach for primary and secondary prevention of CVD based on the available evidence.

## 1 Introduction

Cardiovascular disease (CVD) is a leading cause of death and disability, accounting for approximately one-third of all deaths globally with 19.05 million fatalities in 2020 (1). The burden of CVD disproportionately impacts low- and middle-income countries. For instance, the incidence of CVD in middle-income European Society of Cardiology member countries is estimated to be 30% higher than in high-income countries, yet the resources available to tackle this issue are frequently limited (2, 3). According to the latest clinical guidelines for CVD, current control mainly focuses on lifestyle management, medication and revascularization. Although some medicines are effective in reducing all-cause death, a significant proportion of patients remain at high risk of cardiovascular events, and there is still much room to improve efficacy and reduce adverse effects (4, 5). Given that the current demand for CVD control has not been met, clinicians are considering the potential role of traditional and natural medicines in CVD prevention and treatment (6).

Traditional Chinese Medicine (TCM) has been developed and used continuously for more than 2,000 years and has become increasingly popular in Asia and Western countries over the past few decades, especially acupuncture (7). At present, the integration of traditional Chinese and Western medicine has become China's unique medical system and the most commonly accepted treatment approaches by the Chinese public (8, 9). Past reviews of TCM for CVD have shown that TCM was associated with significant improvements in surrogate end points for hypertension, coronary heart disease (CHD), cardiac arrhythmias, and heart failure. Some Chinese patent medicines could help prevent cardiovascular risk factors and might be used as complementary and alternative approaches for primary and secondary prevention of CVD. However, only a few previous studies have included reports of adverse cardiovascular events and cardiovascular death (6, 10–12). Due to the late development of RCT designs, previous studies are generally of low quality, and their conclusions are not sufficiently reliable or convincing (13).

With the publication of TCM Consort and acupuncture standardized design, reports of high-quality RCTs have gradually increased in recent years (14, 15). TCM follows an ancient physiological system, and believes that health is the result of harmony between body functions and between the body and nature (16). A recent study published in Nature clarified the neuroanatomical basis of the relative specificity of acupuncture points by showing that stimulating different acupoints can activate their respective neural pathways, thus providing preliminary evidence for the concepts of acupoints and acupuncture (17). A review published in the British Medical Journal in 2022 showed that acupuncture has significant effects on 8 diseases, but it is largely absent from clinical practice and policy considerations (18). In recent years, a series of high-quality acupuncture studies have demonstrated the efficacy of acupuncture in digestive, urinary system, and cancer pain (19–22). Similarly, some studies for CVD such as stable angina pectoris, hypertension, myocardial ischemia, and stroke have been gradually published (23–26). In this study, we examine the available evidence from RCTs on the effects of TCM in patients with CVD over the past two decades. We further appraised past RCTs by means of modified Jadad scores, retained high-quality studies, incorporated the most recent high-quality trials, and explored primary outcome events.

## 2 Search strategy and selection criteria

Relevant studies were identified by searching for papers published from January 2004 to January 2024 in MEDLINE (Ovid); EMBASE; the Cochrane Library; Global Health, International Pharmaceutical Abstracts; the China National Knowledge Internet; and the China Biology Medicine, Wanfang, and VIP databases. We also considered the Chinese Clinical Trials Registry (www.chictr.org.cn) and the US Clinical Trials Registry (clinicaltrials.gov).

The search algorithm for MEDLINE was as follows: (“traditional Chinese medicine” OR “traditional Chinese medication” OR “TCM”) AND (“cardiovascular disease” OR “hypertension” OR “blood pressure” OR “angina” OR “coronary heart disease” OR “coronary artery disease” OR “myocardial infarction” OR “stroke” OR “heart failure”) AND (“randomized” OR “randomized controlled trial”) AND “blind,” with no restriction on subheadings. The reference lists of all retrieved papers were checked for other potentially relevant citations, and studies not included in the electronic sources mentioned previously were searched manually. Authors of identified papers will be contacted for further information if necessary.

We included reports of clinical studies with the following criteria: (1) study patients with a definite diagnosis of essential hypertension, myocardial infarction, angina pectoris, coronary artery disease (CAD), stroke or heart failure who were randomized to receive TCM, contemporary medication, or placebo; (2) total sample size ≥ 60 cases; (3) follow-up in each study group ≥ 4 weeks; and (4) quantitative measurements of surrogate endpoints and/or adverse cardiovascular events and/or adverse drug effects available to facilitate outcome analysis. We excluded reports of studies with the following features: (1) studies were nonrandomized and/or non-blinded; (2) patients enrolled had no definite diagnosis; (3) studies compared different TCM medications; and (4) studies reported only symptomatic changes of patients, without objective laboratory measurements or physical examination. (5) methodological quality was evaluated for each study with a Modified Jadad score between 0 (weakest) to 7 (strongest), as described previously; any study with a score < 4 was considered to be of poor quality and was excluded. In addition, when 2 papers reported the results of the same study, the paper with less data was excluded.

Finally, we included 55 eligible reports of RCTs: 39 in English and 16 in Chinese. The time of publication of these 55 studies ranged from 2004 to 2024. Overall, there were 13 reports of hypertension, 21 of CHD, 10 of stroke, and 11 of heart failure.

## 3 Hypertension

High blood pressure currently affects more than 1 billion people worldwide. Hypertension is associated with increased risk of CVD events (coronary heart disease, heart failure, and stroke) and death. The consensus for first-line treatment of hypertension is lifestyle changes, including weight loss, dietary sodium reduction and potassium supplementation, healthy eating patterns, physical activity, and limited alcohol consumption (27). At present, many patients still have uncontrolled blood pressure (BP) due to poor compliance, which increases the risk of cardiovascular disease (28, 29). Risk factors for poor adherence are multifactorial and include side effects of antihypertensive medications and hypertension-related symptoms (30–32). Chinese medicine treats hypertension by identifying the patient's symptoms and physique to develop a personalized treatment plan.

We included 13 randomized controlled trial studies on TCM and essential hypertension, including 10 studies on Chinese patent medicines and 3 studies on acupuncture. The sample sizes ranged from 120 to 628 participants, and the mean follow-up ranged from 4 weeks to 52 weeks. The methodological quality of the included studies was generally high: 9 of 13 reports had a modified Jadad score of 7, 2 of 13 reports had a score of 6, 1 report had a score of 5, and 1 trial on the *Xuemaikang* capsule had a score of 4 (Supplementary Table S1).

**Figure 1:**
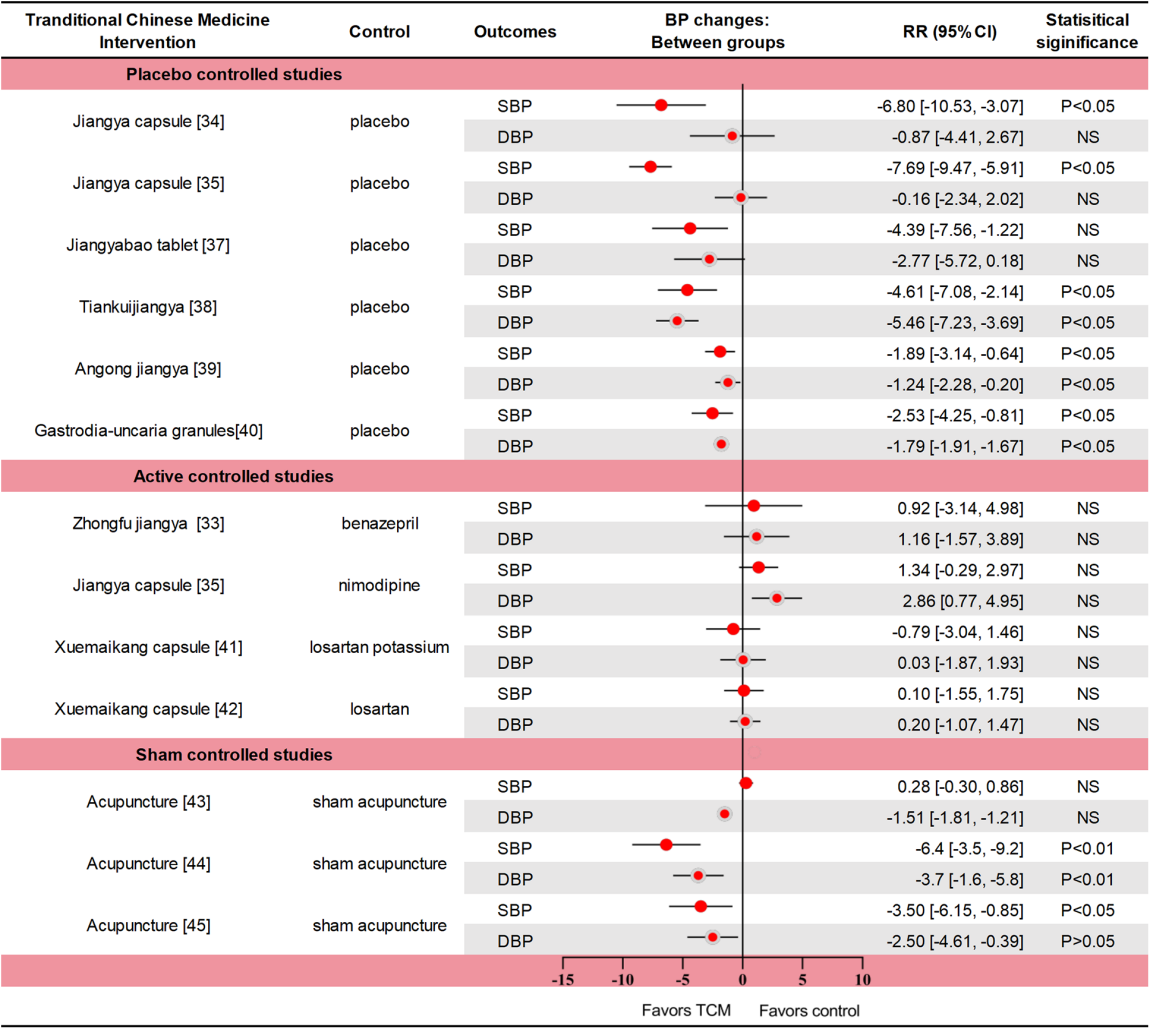
Data summaries From the high-quality RCTs of TCM interventions for hypertension. Numbers in parentheses=reference numbers. BP, blood pressure; CI, confidence interval; DBP, diastolic blood pressure; NS, not significant; RCT, randomized controlled trial; RR, risk ratio; SBP, systolic blood pressure; TCM, traditional Chinese medicine.

The intervention group was treated with *Zhongfu jiangya* capsule, *Jiangya* capsule, *Qiqilian* capsule, *Jiangyabao* tablet, *Tiankuijiangya* tablet, *Gastrodia-uncaria* granules, *xuemaikang* capsule, *Angong jiangya* pill, and acupuncture. Data summaries for all high-quality RCTs of TCM interventions for hypertension are listed in Figure 1.

A multicenter RCT (*n* = 418) compared *Zhongfu jiangya* capsule with Benazepril. The change of blood pressure in the two groups was similar (*p* = 0.661 for SBP and *p* = 0.409 for DBP). In the Zhongfu jiangya group, the 24-hour mean SBP and DBP decreased by 10.67 mm Hg and 7.49 mm Hg from baseline (*p* < 0.01) (33). In a RCT, *Jiangya* capsule significantly reduced SBP (*p* < 0.05) but did not reduce DBP (*p* > 0.05) compared with placebo (34). In another comparison with placebo, nimodipine, *Jiangya* capsules reduced 24-h average SBP and day-average DBP compared with placebo (both *p* < 0.05), but not DBP or night-average SBP (both *p* > 0.05). The therapeutic effect of *Jiangya* capsules and nimodipine were similar (both *p* > 0.05), and the adverse effects were also similar (35). The rate of effective response (defined as a reduction ≥ 10 mmHg in DBP or to the normal range and/or a reduction ≥ 30 mmHg in SBP) to treatment with *Qiqilian* capsule was higher than with placebo (*p* < 0.05) (36). Although the effective response to *Jiangyabao* tablet treatment during the daytime did not differ significantly from that of placebo under standard antihypertensive treatment (*p* > 0.05), both SBP and DBP were significantly lower with *Jiangyabao* than with placebo at night (both *p* < 0.05) (37). After 8 weeks of *Tiankuijiangya* tablet treatment, morning SBP and DBP were reduced by 17.64 mm Hg and 11.85 mm Hg from baseline, respectively, showing statistical significance compared with placebo (*p* < 0.05 for both comparisons). The incidence of adverse reactions was 3.13% (5/160) in the *Tiankuijiangya* group compared to 2.50% (2/80) in the placebo group (38). Liver-fire hyperactivity syndrome is one of the TCM syndroms of hypertension, mainly accompanied by headache (especially both temples), dizziness, irritability, and chest tightness symptoms. In one study (*n* = 338), the antihypertensive effective rate of *Angongjiangya* group was 65.68% (the range of BP was 80%∼120%) after four weeks, and the SBP and DBP were decreased by 9.33 mmHg and 5.57 mmHg (*p* < 0.01), the incidence of adverse events was 1.48% and 3.25%, respectively (39). In a trial of 251 patients with masked hypertension, daytime SBP/DBP was reduced by 5.44/3.39 and 2.91/1.60 mmHg in the *Gastrodia-uncaria* granules and placebo groups (*p* < 0.05). The between-group difference in BP reductions was significant for the daytime (2.52 vs. 1.79 mmHg; *p* ≤ 0.025) and 24-h BP (2.33 vs. 1.49 mmHg; *p* ≤ 0.012), but not for the clinic and nighttime BPs (*p* ≥ 0.162). Only 1 adverse event (sleepiness during the day) was reported (40). One trial compared the efficacy of *Xuemaikang* (SXC) and losartan potassium in the treatment of primary hypertension (*n* = 276). After 8 weeks, the SBP decreased by 10.8 mmHg and 10.01 mmHg, and the DBP decreased by 8.36 mmHg and 8.39 mmHg, respectively (*p* < 0.05) (41). In another RCT of SXC vs. losartan in the treatment of mild hypertension (*n* = 628), DBP changes were similar after 8 weeks of treatment with two groups, decreasing by 7.9 mmHg and 8.1 mmHg respectively. In total cholesterol, SXC group had a significant decrease compared with losartan group, which was −0.1 mmol/L and 0.1 mmol/L (*p* = 0.025). Adverse events during follow-up were similar in both groups (42). Thus, SXC might be an alternative for mild hypertension, particularly for patients with a preference for natural medicine.

The SHARP study (*n* = 192) used the principles of TCM acupuncture to perform active or invasive sham acupuncture for 6 weeks. Active acupuncture was divided into personalized acupuncture and standardized acupuncture, and the treatment effects were similar. The average blood pressure reduction between active and sham acupuncture was not significantly different after 10 weeks (all *p* > 0.05). Acupuncture also showed no advantage over sham acupuncture after 12 months (43). Another acupuncture study (*n* = 140) showed a significant difference in blood pressure after 6 weeks of treatment between the active and sham acupuncture groups, with 24-h SBP and DBP differences of 6.4 mm Hg and 3.7 mm Hg, respectively (*p* < 0.01). In the active acupuncture group, mean 24 h after treatment dynamic SBP and DBP decreased significantly by 5.4 mm Hg and 3.0 mm Hg, respectively. No study-related adverse events occurred (44). In an acupuncture for mild hypertension trial (*n* = 415), personalized acupuncture treatment was similar to standard acupuncture treatment. Compared with the sham acupuncture and the waiting-list group, patients in the active acupuncture group had more reduction in SBP at week 6 (7.2 vs. 4.1 vs. 4.1 mm Hg, *p* > 0.05); However, acupuncture was superior to sham acupuncture (*P* = 0.035) and waiting-list control (*p* < 0.001) at week 9. A total of 9 mild adverse events were reported, most of which were local hematoma and one case of nausea (24).

The theory and practice of acupuncture has been practiced in China for more than 2,000 years, treating diseases by needling specific points on the human body. In fact, the mechanism by which acupuncture affects blood pressure is not well understood. The specific mechanism may be related to the regulation of renin-angiotensin-aldosterone system (RAAS), metabolic disorders, central nervous system, oxidative stress, inflammatory response and vascular endothelial function. In previously published guidance on alternative approaches of reducing blood pressure, acupuncture works similarly to some relaxation techniques (Class III, Level of Evidence B) (45). In recent years, the quality of trials on acupuncture intervention for hypertension has been low, and the conclusions are not sufficiently convincing and can only be regarded as a possible potential benefit (46–48). In the three studies we included, there was insufficient evidence that acupuncture significantly reduced blood pressure.

Current evidence shows that some Chinese patent medicines have antihypertensive effects and a good safety profile, and may be used as an alternative approach. As far as the available clinical evidence is concerned, acupuncture for the treatment of hypertension is still in the early stage of exploration. Differences in the efficacy between personalized (meridian) and standard (non-meridian) acupuncture in treating hypertension are not well understood. Additionally, differences in acupoint specificity and clinical efficacy remain unclear. A total of six trials reported adverse effects or events (all *p* > 0.05); however, long-term effects on blood pressure and cardiovascular (CV) events were less reported.

## 4 CHD

We included 21 RCTs on TCM and coronary heart disease, including angina pectoris, myocardial infarction (MI), CAD, acute coronary syndrome (ACS), and stable myocardial ischemia. 16 of 21 reports had a Modified Jadad score of 7, 2 of 21 reports had a score of 6, 3 of 21 reports had a score of 5. Sample sizes ranged from 66 to 4,870, and follow-up periods ranged from 4 weeks to 4.5 years (Supplementary Table S2).

The Chinese patent medicines in the study included *Danlou* tablet, *Wufu xinnaoqing* capsule, *Shenzhuguanxin* capsule, *Guanxin shutong* capsule, *Xuefuzhuyu* capsule, *Shenmai* capsule, *Xuezhikang* capsule, *Tongxinluo* capsule, *Qishenyiqi* dropping pills, *Xiongshao* capsule, *Qingxin jieyu* tablets, *Yugengtongyu* capsule, *Guanxin danshen*, *Suxiao jiuxin* pills and *Shexiangbaoxin* pills. In addition, several acupuncture trials are covered, including stable angina and ischemic heart disease.

### 4.1 Angina pectoris

The most common clinical manifestation of CHD is angina pectoris and its alleviation is a vital component in the management of patients with CHD. Stable angina is defined here as symptoms that may be attributed to myocardial ischemia, for example, chest discomfort, but lack the duration and severity that one may associate with acute myocardial infarction. The current aim of pharmacologic management of stable angina is to prevent myocardial ischemia episodes, control symptoms, improve quality of life, and prevent cardiovascular events (49). Because of limited medical resources and lack of significant improvement in angina relief from percutaneous coronary intervention, Chinese clinicians choose traditional Chinese medicine and acupuncture in addition to antianginal treatment for chronic stable angina (CSA). Acupuncture in China has been used as non-pharmacological treatment for several decades, especially to relieve symptoms of myocardial ischemia, improve cardiac function, and prevent recurrence (50–57).

In an RCT (*n* = 66), *Danlou* tablet compared with placebo reduced angina pectoris (Phlegm and Stasis Mutual Obstruction Syndrome) attack frequency, pain duration, consumption of nitroglycerin and the level of hs-CRP was decreased (all *p* < 0.05) (58). On the basis of conventional treatment, 240 patients with chronic stable angina pectoris were treated with *Wufuxinnaoqing* capsule and placebo. After 12 weeks, the total effective rate of the treatment group, the rate of nitroglycerin withdrawal and reduction was superior to that of the control group (all *p* < 0.01) (59). In a RCT (*n* = 187) comparing *Shenzhuguanxin* capsule with placebo after 12 months, APS was reduced by 76.7% in treatment and 53.8% in placebo group, and CM symptom scores were reduced by 18.3% and 16.1%, respectively (60).

One RCT (*n* = 232) that *Xinlin* pills significantly increased the total duration of treadmill exercise after 4 weeks (*p* < 0.05) by FAS analysis. In addition, the reduction of nitroglycerin dose was 2.45 tablets and 0.5 tablets per week (*p* < 0.05), the frequency and duration of angina pectoris attacks were also significantly reduced (*p* < 0.05) (61). A total of 4 weeks of treatment with *Guanxinshutong* (GXST) resulted in a statistically significant lowering of the time of angina attacks and the consumption of nitroglycerin dose (*p* < 0.01). The incidence of adverse events was similar between the two groups (*p* = 0.17). Five adverse events (AEs) were reported in the GXST group, all of which were gastrointestinal disturbances (62). In a small sample (*n* = 90) RCT of unstable angina pectoris after PCI, compared with placebo, *Xuefuzhuyu* granule had a lower BSS score after 4 weeks (*p* < 0.01), and were beneficial in terms of physical pain, angina stability, and angina pectoris (*p* < 0.05) (63). The three trials had low rates of adverse events and reactions, and good safety profile. Another trial of 324 patients with stable angina from 13 hospitals in China evaluated the efficacy of *Suxiao jiuxin* pills. Compared with the control group, the curative efficacy rate of stable angina, the curative efficacy rate of TCMSS significantly increased, and the total score of angina pectoris symptoms and TCMSS significantly reduced in the *Suxiao jiuxin* pills group, accompanied by the statistically significant improvement in the curative efficacy rate based on CCS grade reduction (all *P* < 0.05). The medication compliance, concomitant medication, and rates of adverse events were similar between the two groups (*P* > 0.05) (64).

In the 2019 high-quality acupuncture trial for stable angina pectoris, 398 participants (mean age = 62.6 years) were divided into four groups (DAM: Disease & Meridians; NAM: non-disease affecting meridian points; SA: false acupuncture; WL: not receiving acupuncture), after 16 weeks of intention-to-treat, compared with other group, acupuncture on the DAM as adjunctive treatment to antianginal therapy showed superior benefits in alleviating angina (*p* < 0.01). 16 patients reported mild to moderate adverse events related to acupuncture, which did not require medical intervention (23). Due to the complexity and difficulty of the acupuncture trials, the large sample of nearly 400 people did not show significant side effects, thus, the superior benefits of acupuncture in alleviating angina are considered highly convincing.

In these RCTs of angina pectoris, TCM interventions including *Danlou* tablet, *Wufuxinnaoqing* capsule, *Shenzhuguanxin* capsule, *Xinlin* Pill, GXST capsule, *Xuefuzhuyu* granule and acupuncture treatment had positive effects on angina pectoris attack frequency and nitroglycerin consumption during a follow-up period ranging from 4 to 52 weeks. Six studies reported adverse effects, two of which reported cardiovascular events. Compared with placebo, *Wufuxinnaoqing* and *Shenzhuguanxin* could effectively decrease cardiovascular events. Furthermore, based on Eastern acupuncture theory, the incidence of angina pectoris in the DAM group (Disease & Meridians) was lower than that in the NAM group (non-disease affected meridian acupoint group), and there were no adverse CV events during the treatment.

### 4.2 Myocardial infarction

Myocardial infarction is also a major life-threatening condition worldwide. Despite reperfusion therapy and optimal medical management, patients with STEMI still face high risks of inhospital mortality and recurrent cardiovascular events (65–67). Tongxinluo was initially evaluated and approved in China for angina pectoris and ischemic stroke in 1996 (68). After that, some Chinese patent medicines were gradually used for acute coronary syndrome (ACS).

The 2008 CCSPS trial evaluated *Xuezhikang* for MI with major adverse CV events as the endpoint. They were randomly assigned to receive daily treatment with *Xuezhikang* (*n* = 2,429) or placebo (*n* = 2,441) and were followed for an average of 4.5 years. The primary endpoint included CV death and non-fatal MI. The incidence of the primary CV events was significantly lower with *Xuezhikang* treatment than with placebo (*p* < 0.05). Serum total cholesterol, low-density lipoprotein cholesterol and triglyceride levels in the *Xuezhikang* group were lower than those in the placebo group (*p* < 0.001). *Xuezhikang* was deemed safe and well tolerated, with no treatment-related serious adverse events reported (69).

One study (*n* = 219) showed that *Tongxinluo* capsule reduced myocardial non-reflow and infarct size after acute MI direct PCI, improved myocardial perfusion. *Tongxinluo* significantly improved ST-segment recovery 6 h after reperfusion (*p* < 0.05) and lasted until 24 h after reperfusion (*p* < 0.01) (70). In another recent study on Tongxinluo, published in JAMA in 2023, researchers conducted a 12-month randomized, double-blind, placebo-controlled trial in China in patients (*n* = 3,797, mean age = 61.1 years) who developed STEMI within 24 h. The primary endpoint was Major Adverse Cardiovascular and Cerebrovascular Events (MACCEs), including cardiac death, myocardial reinfarction, emergency coronary revascularization, and stroke. MACCEs occurrence was lower in the *Tongxinluo* group than in the control group during the 30-day and 1 year period (both *p* < 0.01), Cardiac death was also lower than in the placebo group (both *p* < 0.05). Adverse drug reactions were more frequent in the *Tongxinluo* group compared to the placebo group (2.1% vs. 1.1%, *p* = 0.02), mainly driven by gastrointestinal symptoms. The trial demonstrated that *Tongxinluo* can be used as a supplement to STEMI guidelines, significantly alleviating clinical outcomes at 30 days and 1 year with mild adverse effects (71).

One large RCT (*n* = 3,505) compared the efficacy and safety of *Qishenyiqi* (QSYQ) dropping pills with aspirin in secondary prevention of myocardial infarction. The results showed that QSYQ had similar effects to aspirin and less adverse reactions. The incidence rates of primary outcomes at 12 and 18 months was 2.98% and 3.67% for the QSYQ group and 2.96% and 3.81% for the aspirin group, respectively. Cardiovascular death and mild adverse effects were similar between the two groups (*p* > 0.05) (72). Eighty-three MI patients were randomly assigned to receive either Danlou tablets or placebo for 90 days of treatment. *Danlou* tablet could significantly reduce left ventricular end-diastolic volume index and left ventricular end-systolic volume index (both *p* < 0.01), and increase left ventricular ejection fraction (*p* < 0.01). In terms of safety, the incidence of major adverse cardiovascular events in the *Danlou* group was lower compared with placebo (*p* < 0.05), and the composite incidence of two groups was 11.9% and 34.15%, respectively (73).

MI studies have shown that TCM could effectively improve hard endpoints and/or surrogate endpoints, while its adverse effects are acceptable. Four studies reported adverse cardiovascular events and composite endpoints, especially in the three large-sample RCTs, *Xuesaitong*, *Tongxinluo* and QSYQ were proven to reduce cardiovascular events and all-cause mortality.

### 4.3 CAD

Either incidence or mortality of coronary artery disease (CAD) accounts for a large part of cardiovascular diseases (74–76). According to the latest ESC clinical practice guidelines, and the treatment is mainly based on lifestyle management, medical treatment, and revascularization (77). However, despite the use of medical treatment and revascularization, there is still much room for improving efficacy and reducing adverse reactions (5). In China, TCM is a potential add-on treatment and has a long history being used for the treatment of CAD (6, 78).

In one RCT, patients with restenosis after PCI were randomized to receive *Xiongshao* or placebo, 6 months of treatment with *Xiongshao* ameliorated restenosis (*p* < 0.05) and decreased cardiovascular events with borderline statistical significance (*p* = 0.051) after PCI, compared with placebo without any drug-related adverse events (79). In a multicentre, double-blind, randomized trial, patients with stable CHD (*n* = 1,500, mean age = 60.3 years) were randomly assigned to receive *Qingxin jieyu* capsules or placebo for 6 months. The primary outcomes were similar between the two groups (*p* > 0.05). However, the absolute risk of composite “hard” endpoints was reduced by 0.99% in *Qinxin jieyu* groups (80). In one RCT involving 114 patients with CHD, the incidence of the comprehensive outcome of *Yugengtongyu* granules was significantly lower (*p* < 0.05). The primary outcomes and independent events such as non-fatal MI showed a decreasing trend. A total of 11 adverse events occurred at one year of follow-up, the most common event being constipation (81).

Two hundred CHD patients with depression and anxiety after PCI were included and randomly assigned to receive Guanxin Danshen or placebo. After 12 weeks of treatment, the PHQ-9 and GAD-7 scores in the *Guanxin danshen* were significantly lower than those in the control group (*p* < 0.05), and the incidence of MACE in the two groups was similar (*p* > 0.05) (82). A multicenter RCT investigated the safety and efficacy of *Suxiaojiuxin* pills, the most frequently used drug in the cardiovascular acute phase in China, in patients with ACS with early PCI (*n* = 200, mean age = 61.2 years). The occurrence of MACE in *Suxiaojiuxin* Pills group was lower than that in placebo group (*p* < 0.05). In addition, *Suxiaojiuxin* pills might improve patients' LVEF levels and SAQ scores during long-term follow-up (83). 151 patients (mean age = 63 years) with stable ischemic heart disease were randomized to TA (tranditional acupuncture), sham acupuncture and waiting control. For markers of parasympathetic tone, withdrawal stress HRV was higher in the TA group than in the SA group (*p* < 0.05), with vagus activity 17% higher (*p* = 0.008) (54).

MUSKARDIA trail included 2,674 patients with stable CHD randomly received *Shexiangbaoxin* pills and placebo, the incidence of MACE at was 1.9% and 2.6% after 24 months. *Shexiangbaoxin* pills had a 26.9% reduction in the incidence of MACE compared to the placebo group. Angina frequency was significantly reduced in the *Shexiangbaoxin* group at 18 months (*p* = 0.0362). Other secondary endpoints and adverse events were similar between the two groups(both *p* > 0.05) (84). Another study on the MUSKARDIA trial evaluated the efficacy and safety of patients with stable CHD and diabetes mellitus (DM or FBG ≥ 7.0 mmol/L). Compared with placebo groups, *Shexiangbaoxin* pills were significantly lower in the incidence of MACE (*p* < 0.05) and secondary composite outcomes (*p* < 0.05). In patients with uncontrolled DM (≥ 4 measurements of FBG ≥ 7 mmol/L in five times of follow up), the risk of secondary outcomes was also significantly lower than in the placebo group (*p* < 0.05) (85).

In these CAD trials, 7 studies reported similar adverse effects within the groups. *Xiongshao* capsule, *Qingxin jieyu* capsule, *Xinkeshu*, *Yuyuetongyu*, *Guanxin danshen*, and acupuncture have certain effects on improving CAD-related surrogate endpoints. *Qingxin jieyu* capsule *and Suxiaojiuxin* pills demonstrated significant advantages in reducing cardiovascular (CV) events (*p* < 0.05).

## 5 Stroke

Stroke, characterized by high morbidity, disability, and mortality, is the leading cause of death in China, contributing substantially to the global burden of diseases (86). The disease burden of stroke is still severe in China, although the age-standardised incidence and mortality rates have decreased since 1990 (87).

Currently, recanalization therapy via intravenous thrombolysis and mechanical thrombectomy has allowed a great breakthrough in improving the neurologic prognosis of patients with ischemic stroke (88). However, recanalization therapy benefits a limited number of patients because of the strict time window and high operational requirements (8). Therefore, the discovery and development of some effective and safe alternative therapies are needed to further improve the prognosis of patients with ischemic stroke.

A total of 10 RCTs on TCM and ischemic stroke were included, including 1 study on acupuncture. Sample sizes ranged from 100 to 2,966, and follow-up periods ranged from 4 to 102 weeks. The modified Jadad scores of the included studies were all >5, 7 of 10 reports had a score 7, 2 of 10 reports had a score 6, 1 of 10 report had a score 5. (Supplementary Table S3). The intervention group was treated with *Naoxinduotai* capsule, *Sanqitongshu* capsule, acupuncture, *Ginkgo* tablet, *Neuroaid*, *Dihuangyinzi*, *YangyinYiqi Huoxue* granule*, Xuesaitong* capsule. The control group received placebo and sham acupuncture.

Compared with placebo, *Naoxinduotai* capsule could significantly improve nerve function and Barthel index in ischemic stroke (*p* < 0.05), and no drug-related adverse reactions were found in the Naoxinduotai group (89). An RCT included 605 ischemic stroke patients who were given *Danqi Piantang* (DJ) capsules or placebo for 1 month. Functional outcome was measured by the Stroke Diagnosis and Treatment Effect Scale's comprehensive function score, which showed that the DJ group was better than the control group (*p* < 0.05), its adverse effects and tolerability were acceptable (90). 140 patients with anterior circulation ischemic stroke within 30 days of onset were randomly treated with *Sanqitongshu* capsules or placebo in addition to aspirin treatment. *Sanqitongshu* capsule could significantly improve the neurological deficit score and BI score (both *p* < 0.05). The adverse reactions manifested by gastrointestinal discomfort were similar (91). 290 patients with initial acute ischemic stroke (≥24 h but within 14 days) were randomly divided into acupuncture group and control group (sham acupoints) on the basis of standard treatment. There were significant differences in mean BI and quality of life scores between the two groups at 6 months (both *p* < 0.01). The NIHSS scores of the two groups showed no significant differences at 2 weeks (*p* > 0.05), but there was a significant difference at 4 weeks (*p* < 0.01). In terms of recurrent stroke, 6 cases in the acupuncture group and 34 cases in the control group (*p* < 0.01) (25). Patients with acute ischemic stroke (*n* = 102) were randomized to receive *Ginkgo* tablet and placebo for 4 months. At 4-month follow-up, the NIHSS score in the *Ginkgo* tablet group decreased by 50% from baseline, significantly more than the placebo group (*p* < 0.05) (92).

In an RCT of the efficacy of Chinese medicine *Neuroaid* in stroke recovery, MLC601 was statistically no better than placebo in improving outcomes at 3 months when used among patients with acute ischemic stroke of intermediate severity (93). The CHIMES-E trial (*n* = 880, mean age = 61.8 years) was designed to evaluate the effect of *Neuroaid* treatment on long-term outcomes in subjects with cerebral infarction (moderate severity within 72 h) after 3 months of treatment. The likelihood of achieving functional independence was significantly increased after 6 months of treatment with *Neuroaid* compared with placebo and the benefits continued until 18 months after stroke. However, the outcomes at 24 months were similar. In addition, overall mortality and incidence of vascular events were also similar between two groups (both *p* > 0.05) (94). One hundred patients with ischemic stroke (less than 30 days) were randomly assigned to receive *Dihuangyinzi* or placebo for 12 weeks. The Fugl-Meyer score and BI in *Dihuangyinzi* group were significantly improved compared with those in the placebo group (*p* < 0.05) (95). An RCT (*n* = 288) evaluated the efficacy of *Yangyin Yiqi Huoxue* granule (YYHG) in treating Ischemic Stroke with Qi-Yin Deficiency and Blood Stasis Syndrome. The comprehensive cure rates in the high-dose YYHG treatment group were significantly higher than in the other three groups (*P* < 0.01). The improvement of NIHSS, ADL, QLI, and CMS scores in both the high-dose and low-dose YYHG groups was significantly superior to that of the positive control group and the placebo control group (*P* < 0.05). Regarding safety, adverse reactions after YYHG treatment were generally mild (3.78%), and no serious adverse reactions were reported (96). A large-scale RCT included 2,966 Chinese adults with ischemic stroke, treated with *Xuesaitong* capsule (*Panax notoginseng Saponins*) and placebo; At 3 months, *Xuesaitong* capsule significantly increased the likelihood of functional independence in ischemic stroke patients (*p* < 0.01). Serious adverse events were similar between the two groups (*p* > 0.05). This study suggests that *Xuesaitong* is a safe alternative therapy for improving the prognosis of ischemic stroke (97).

In these RCTs, two studies reported on the primary outcome; the outcome events and adverse events of TCM intervention were similar to those of the control group (*P* > 0.05); One of the large-sample, high-quality trials showed that *Xuesaitong* was slightly lower than the placebo group in terms of cardiovascular events and mortality, but there was no significant difference (*p* > 0.05).

## 6 Heart failure

Ischaemic heart disease has become the main cause of heart failure (HF) in China in recent decades (98, 99). Patients with ischaemic HF (IHF) have a poorer quality of life and worse prognosis than those without IHF, even after receiving the standard international guideline-directed medical therapy. Traditional Chinese medicine may have a complementary effect in improving exercise tolerance and cardiac function among patients with HF (100–104). It has been reported that TCM may have complementary effects in improving exercise tolerance and cardiac function in patients with heart failure.

We included and evaluated 11 RCTs of TCM and heart failure (HF). Sample sizes ranged from 80 to 3,110 patients, and follow-up periods ranged from 4 weeks to 36 months. 9 of 11 reports had a Modified Jadad score of 7, 2 of 11 reports had a score of 6 (Supplementary Table S4). The intervention group was treated with *Nuanxin* capsule, *Shencaotongmai* granule, *Qiangxintongmai*, *Qiliqiangxin*, *Zhuanshenling*, *Qishenyiqi, Shensongyangxin* granule, *Qishen* granules, *Buyanghuanwu*. Standard treatment was based on clinical guidelines for HF, and all control groups received placebo.

In one RCT of 150 patients with chronic HF, based on standard treatment, the total effective rate was 85.9% in *Nuanxin* capsule group and 63.0% in control group. The decrease in NYHA score was more significant in the *Nuanxin* group (*p* < 0.01), and it improved heart function in chronic HF with fewer adverse reactions (105). A total of 280 patients with chronic HF (NYHA classes II and III) were randomized to receive *Shencaotongmai* granules or placebo for 12 weeks. The added value of LVEF in *Shencaotongmai* group was significantly higher than placebo (*p* < 0.05). The incidence of adverse reactions in both groups was 0.71% (106). 280 patients with chronic HF were randomized to *Qiangxintongmai* and placebo. After 12 weeks of treatment, the *Qiangxintongmai* group had more significant improvement in 6-minute walking test and LHFQ score (both *p* < 0.01), no related adverse reactions were reported (107). A RCT was conducted in 140 patients with chronic HF (heart-kidney Yang deficiency). After 12 weeks of external application of *Zhuangshenling* formula, the improvement of BNP level was significantly better than control group (*p* < 0.05). 6-min walking distance between the two groups after treatment was similar (*p* > 0.05), and no adverse events were reported (108). In a RCT (*n* = 411), *Shensongyangxin* granule caused a significantly greater decline in the total number of ventricular premature complexes (VPCs) than the placebo did (*p* < 0.05), The secondary endpoints of the LVEF, NYHA classification, NT-proBNP, 6MWD, and MLHFQ scores showed a greater improvements in the *Shensongyangxin* group than in the placebo group (*p* < 0.05). In this 12-week study, *Shensongyangxin* was demonstrated to have the benefits of VPCs suppression and cardiac function improvement with good compliance on a background of standard treatment for CHF. The analysis of drug-induced adverse events revealed no differences between the two groups (109).

One RCT randomly divided 228 patients with HF (NYHA class of II to III) into two groups, to be treated with Yangxinkang tablet or placebo for 4 weeks. Compared with placebo, scores on the Minnesota Heart Failure Living Questionnaire (MLHFQ) showed a significant improvement (*p* < 0.05), with no treatment-related adverse events (110). A total of 640 HF patients were randomly assigned to receive QSYQ or placebo for six months. Compared with placebo, the QSYQ group had a significant increase in 6-minute walking distance and Minnesota heart failure questionnaire scores at 6 months (both *p* < 0.01). The incidence of the composite endpoint was similar between the two groups at 6 and 12 months (111). In a twelve-week RCT (*n* = 191), the *Qishen* granules group demonstrated a considerably greater reduction in NT-proBNP than the placebo group (*p* = 0.011). Patients who received *Qishen* granules performed better in the NYHA functional rank, 6MWD, TCM syndrome integral scale, and quality of life (*p* < 0.05). The *Qishen* granules performed better in HFrEF patients regarding the efficiency of NT-proBNP (112). A trial has proven that *Buyanghuanwu* treatment can further improve cardiac dysfunction and clinical symptoms of IHF on the basis of standard treatment without obvious adverse reactions. After 3 months of treatment, the NYHA classification, TCM syndrome scores, and the percentage of subjects with at least 30% reduction in NT-ProBNP were significantly improved in the BYHW group, compared with the control group (*p* < 0.05) (113).

A multicenter RCT study evaluated the efficacy of *Qiliqiangxin* capsule in patients with chronic HF (*n* = 512). During the 12-week follow-up, the reduction of NT-proBNP in *Qiliqiangxin* capsule group was significantly greater than that in placebo group (*p* = 0.002); in terms of reducing NT-proBNP by at least 30%, the proportion in the *Qiliqiangxin* capsule group was greater than that in the placebo group (*p* < 0.001). The serious adverse events and drug-related adverse events were similar (*p* > 0.01) (102). The recent QUEST trial (*n* = 3,110) evaluated the clinical efficacy and safety of *Qiliqiangxin* capsule for major heart failure outcomes in patients with HFrEF (LVEF ≤ 40%; NT-pro BNP ≥ 450 pg/ml). During a median follow-up of 18.3 months, the incidence of MACE in *Qiliqiangxin* group was significantly lower than that in placebo group (*p* < 0.001). In terms of secondary endpoints, serum NT-proBNP in *Qiliqiangxin* group decreased significantly more than that in control group during 3 months of follow-up (*p* = 0.047). *Qiliqiangxin* capsule was well tolerated, safety endpoint analysis in two groups showed that was similar (*p* = 0.058) (114).

In summary, these studies suggest that Chinese patent medicines, such as *Qiliqiangxin*, *Nuanxin*, *Shencao tongmai*, *Yangxinkang*, might be effective in improving cardiac remodeling and function in patients with chronic heart failure. Six studies reported similar adverse effects or events, and with safe profile.

## 7 Adverse cardiovascular events and death events

**Figure 2:**
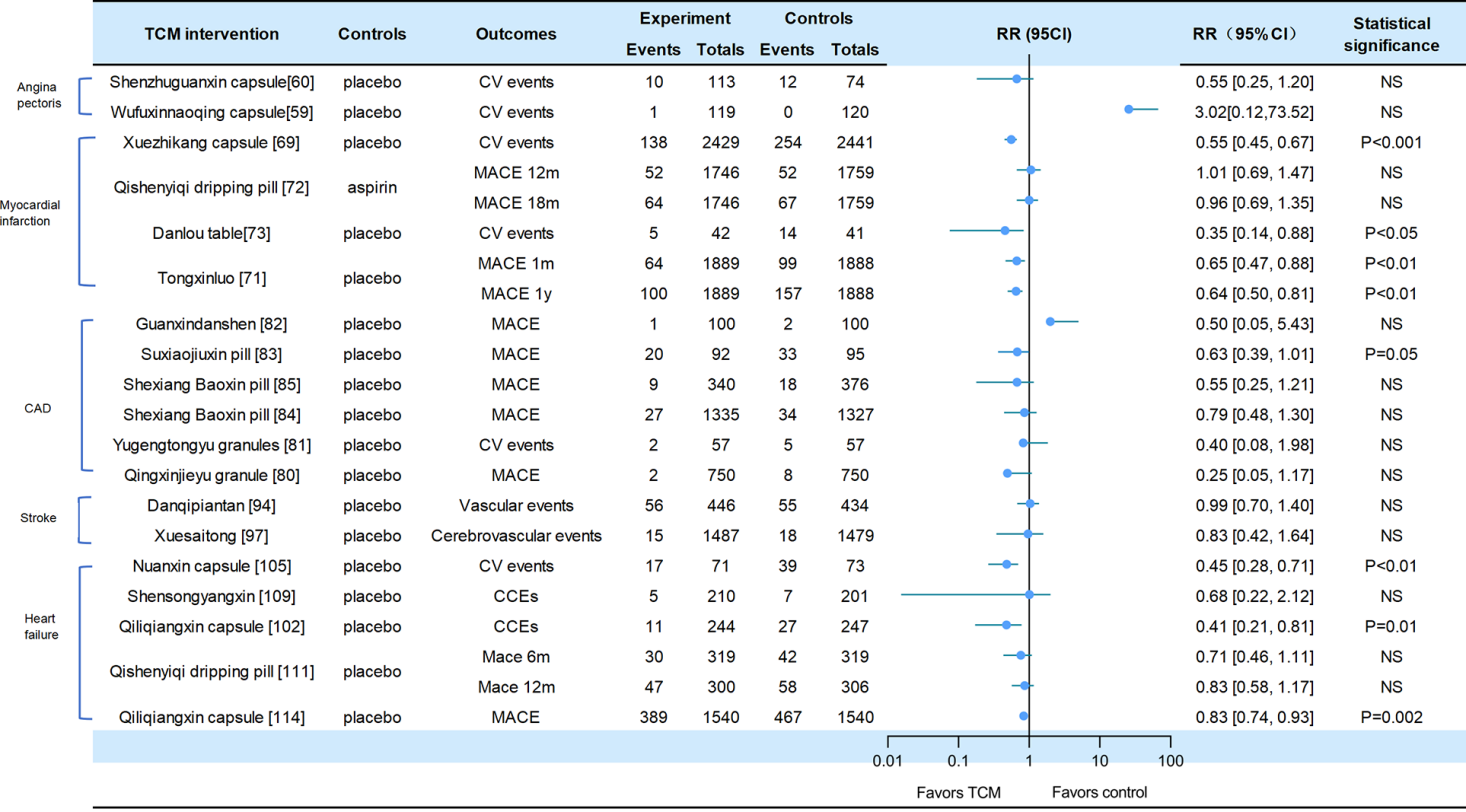
Cv events in traditional Chinese medicine interventions for CVD. Numbers in parentheses=reference numbers. CV, cardiovascular; CVD, cardiovascular disease; TCM, traditional Chinese medicine; NS, not significant; RR, risk ratio.

**Figure 3:**
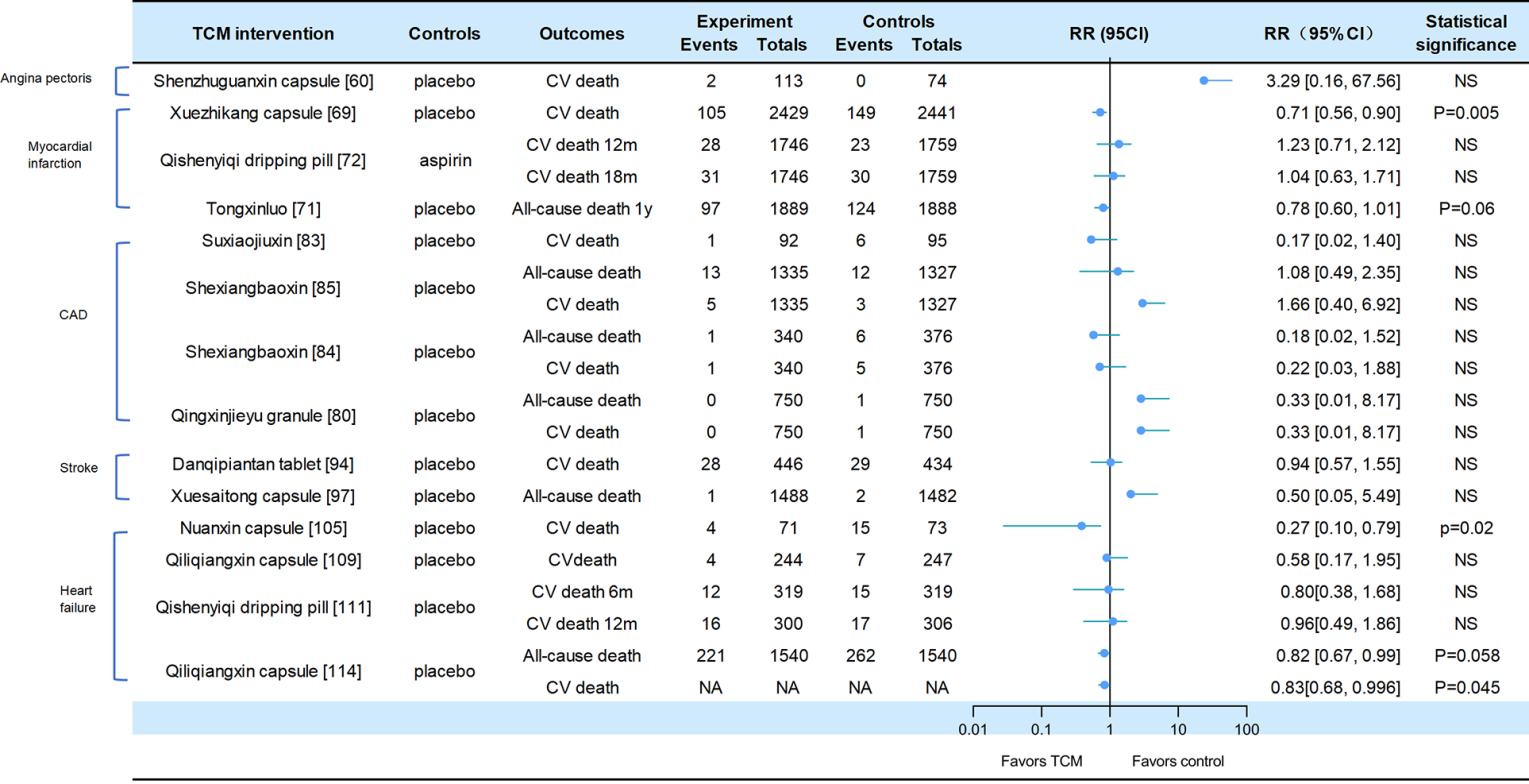
Death events in traditional Chinese medicine interventions for CVD. Numbers in parentheses=reference numbers. CV, cardiovascular; CVD, cardiovascular disease; TCM, traditional Chinese medicine; NS, not significant; RR, risk ratio.

A total of 19 of the included studies reported adverse CV events, all-cause mortality, or CV death, including heart failure, stroke, and CAD (CV events for Figure 2, death events for Figure 3).

Adverse cardiovascular events were reported in two trials of angina pectoris. Compared with placebo, SZGX group had a lower risk of CV event (8.85% [10/113] vs. 16.22% [12/74], *p* > 0.05), and CV death (1.8% [2/113] vs. 0 [0/74], *p* > 0.05) (60). Additionally, *Wufuxinnaqing* showed similar rates of CV events compared to the placebo (0.84% [1/119] vs. 0 [0/120], *p* > 0.05) (59).

Cardiovascular events and mortality were reported in four trials of myocardial infarction. The *Xuezhikang* group had a significantly lower incidence of primary cardiovascular events compared to the placebo group, including major coronary events [5.7% (138/2,429)] vs. 10.4% [254/2,441], *p* < 0.001), and CV death [4.3% (105/2,429)] vs. 6.1% [149/2,441], *p* = 0.005). During the 4.5-year follow-up period, *Xuezhikang* capsule not only reduced cardiovascular mortality and total mortality by 30% and 33%, respectively, but also lowered the risk of non-fatal MI compared to the placebo (*p* < 0.001) (69). In the Shang 2013 trial, the incidence of MACE was similar between *Qishen yiqi* dripping pills and aspirin group at 12 months (*p* = 0.872) and at 18 months (*p* = 0.957). The CV mortality rates were also similar in two groups at 12 months (*p* = 0.411) and at 18 months (*p* = 0.782). The incidence rates of non-fatal MI and non-fatal stroke were also significantly different (*p* > 0.05) (72). In the Mao 2016 trial, *Danlou* had significantly lower CV events (11.90% [5/42] vs. 34.15% [14/41], *p* = 0.02); as well as significantly lower rates of non-fatal MI (11.9% [5/42] vs. 21.95% [9/41]) and severe heart failure (2.38% [1/42] vs. 12.2% [5/41]) (73). In the 2023 trial of *Tongxinluo* for MI, the occurrence of MACCEs was lower in the *Tongxinluo* group than in the control group during the 30-day period (64 [3.4%] vs. 99 [5.2%], *p* < 0.01). Cardiac death was also lower in the *Tongxinluo* group (56 [3.0%] vs. 80 [4.2%], *p* < 0.05). After 1 year, the incidence of MACCE (100 [5.3%] vs. 157 [8.3%], *p* < 0.01) and cardiac death (85 [4.5%] vs. 116 [6.1%], *p* < 0.05) remained lower (71).

Six trials reported cardiovascular events and mortality in CAD. The Wang 2023 trial reported that the incidence of MACE was similar between the GXDS and placebo groups (1% [1/100] vs. 2% [2/100], *p* > 0.05), and all patients experienced cardiogenic rehospitalization (82). The Shen 2020 trial reported a lower incidence of major cardiovascular events in SJP groups compared to placebo group (21.7% [20/92] vs. 34.7% [33/95], *p* < 0.05). The cases of MACE (death/myocardial infarction/stroke/heart failure rehospitalization) in the SJP and placebo groups were 1/2/12/5 and 6/4/9/14, respectively. The analysis showed the SJP group had a lower incidence of MACE than the placebo group (MACE/not MACE, 20/70 vs. 33/62, *p* < 0.05) (83). The Zhou 2023 trial reported that the incidence of MACE (2.6% [9/340] vs. 4.8% [18/376], *p* = 0.192) and all-cause mortality (0.3% [1/340] vs. 1.6% [6/376], *p* = 0.127) was similar between the MUSKARDIA and placebo groups. In addition, there was no significant difference in CV death (0.3% [1/340] vs. 1.3% [5/376], *p* = 0.665) and composite outcomes (15.3% [52/340] vs. 22.6% [85/376]), *p* = 0.192) (85). In the MUSKARDIA subgroup analysis, the incidence of MACE at 24 months was similar in both groups (*p* = 0.2869). Furthermore, there were similar rates in all-cause mortality (*p* = 0.8526) and CV death (*p* = 0.7119). After two years, the incidence of MACE events decreased by 26.9%. The incidence of non-fatal MI (*p* = 0.3656) and no-fatal stroke (*p* = 0.9898) was similar in both groups (84). In Wang 2021 trial, the major outcomes of *Yugengtongyu* granules and placebo were similar (*p* = 0.435). There were no cardiovascular deaths or all-cause deaths in both groups during the follow-up period; compared with placebo, all composite outcomes were lower in *Yugengtongyu* group (*p* = 0.013), but was similar in non-fatal MI [0 vs. 8.77% (5/57), *p* = 0.067] (81). In Li 2019 trial, *Qingxin Jieyu* granule showed a significantly lower incidence of MACE compared to the placebo (*p* = 0.012); the hard endpoint was reduced by 0.99% during the follow-up. Additionally, there were no differences in all-cause mortality [0 vs. 0.16% (1/750), *p* = 0.95] and CV death [0 vs. 0.16% (1/750), *p* = 0.95] (80).

Two trials focused on stroke reported cardiovascular events. In the CHIMES-E trial, vascular events (12.6% [56/446] vs. 12.7% [55/434], *p* > 0.05) and all-cause mortality (6.3% [28/446] vs. 6.7% [29/434], *p* > 0.05) in *NeuroAid* were similar to those in the placebo group (94). In a 2023 study of *Xuesaitong* for stroke, the incidence of cerebrovascular events (1% [15/1,487] vs. 1.2% [18/1,479], *p* = 0.59) and all-cause death (0.1% vs. 0.1%, *p* = 0.56) were similar between two groups (97).

Cardiovascular and mortality events were reported in the five trials of heart failure. The *Nuanxin* group had lower rates of cardiovascular events (23.9% [17/71] vs. 53.4% [39/73], *p* < 0.05) compared to the placebo group; however, cardiovascular death rates were similar in both groups (5.63% [4/71] vs. 8.95% [15/73], *p* > 0.05) (105). The incidence of CCEs in *Shensongyangxin* and placebo groups were 2.2% and 3.5%, respectively (*p* > 0.05) (109). *Qiliqiangxin* showed a lower incidence of CCEs compared to the placebo (4.51% [11/244] vs. 10.93% [27/247], *p* = 0.008), while the mortality rate was similar in both groups (1.64% [4/244] vs. 2.83% [7/247]) (102). In the trial of *QSYQ* for heart failure, the incidence of MACE at 6 months was not significantly lower in *QSYQ* group compared to the placebo (9.40% [30/319] vs. 13.17% [42/319], *p* = 0.255); the incidence of MACE at one year was similar (15.67% [47/300] vs. 19.12% [58/306]) (111). In Li 2023 trial, the incidence of CV death events was similar at six months (3.76% [12/319] vs. 4.70% [15/319], *p* = 0.689) and at one year (5.33% [16/300] vs. 5.64% [17/306], *p* > 0.05); the incidence of composite endpoint events was similar at 6 months (13.17% vs. 16.61%) and 12 months (20.38% vs. 22.88%). Additionally, *Qiliqiangxin* reduced cardiovascular death (*p* = 0.045) (114).

## 8 Conclusions

We initially conducted a search for studies on other cardiovascular risk factors and diseases in recent years, such as hyperlipidemia, hyperglycemia, and arrhythmia. There were only a limited number of valuable studies. For example, the SS-AFRF study, as reported in ESC 2023, also recently published in the European Heart Journal, showed that Shensong Yangxin capsule significantly reduced atrial fibrillation (AF) recurrence after perAF ablation (115). Therefore, we focused on including high-quality studies that have been updated in recent years for our evaluation.

The current evidence from RCTs indicates that Chinese patent medicines could effectively reduce the blood pressure of hypertensive patients, decrease the occurrence of coronary artery events in patients with a history of myocardial infarction (especially with the use of *Xuesaitong*, *Qishenyiqi* pills and *Tongxinluo*), alleviate the severity of angina pectoris and myocardial infarction, and reduce the frequency of angina attacks and consumption of nitroglycerin (especially *Shensong yangxin* capsule and acupuncture). TCM interventions have also shown promise in improving the functional independence of stroke patients (particularly with the use of *Xuesaitong* granules) and enhancing heart function in patients with heart failure (particularly with the use of *Qiliqiangxin* capsule). Furthermore, acupuncture may contribute to the restoration of limb function following a stroke and mitigate the onset of angina pectoris.

Regarding primary outcomes, TCM interventions, compared to placebo or Western medicines, did not increase the risks of CV events or CV death in included trials, except for hypertension trials (which were no mentioned). Some of these TCM interventions, including *xuezhikang*, *Qishenyiqi* pills, *tongxinluo*, *suxiaojiuxin* pills, *qiliqiangxin*, have been found effective in reducing cardiovascular events.

Furthermore, the occurrence of adverse reactions or events did not increase in most trials. It is worth mentioning that our review also has some limitations, some studies with short follow-up periods and small sample sizes, which may result in the long-term prognosis of TCM treatment remaining unclear. Additionally, some of the trials included in our review were published in Chinese, and due to language barriers, this may lead to the findings being easily overlooked by researchers whose native language is English. Of course, in the future, more high-quality randomized controlled trials will be needed to validate our conclusions.
