# Supplementary material for: Traditional Chinese medicine for cardiovascular disease: efficacy and safety
Source: Front Cardiovasc Med. 2024 Dec 3;11:1419169. doi: 10.3389/fcvm.2024.1419169 (PMC11649660; doi:10.3389/fcvm.2024.1419169)
Supplement: Supplementary file 1 [file Table1.pdf]

Supplementary Table S1: Characteristics of the randomized controlled trials of TCM for hypertension

Studies included	Clinical diagnosis	Duration of disease (months)	Patient (n)	Mean age (years)	Treatment comparison	Standard therapy	Follow up (weeks)	Endpoints	BP levels	Efficacy	Safety (Adverse events)	Modified Jadad score
Kou QA, et al. 2007 [33]	Untreated hypertension with SBP > 150 mmHg or DBP >95 mmHg	47.1	418	53.2	*Zhongfujiangya* capsule versus benazepril	None	4	BP; drug-related side effects	Baseline 24-hr SBPs: 140.1 vs. 141.0 mmHg; baseline 24-hr DBPs: 88.9 vs. 88.8 mmHg; follow-up SBPs: 129.6 vs.129.5 mmHg; follow-up DBPs: 81.3 vs. 80.1 mmHg; SBP changes: -10.7 vs. -11.6 mmHg; DBP changes: -7.5 vs. -8.7 mmHg.	Positive	Yes（0 vs. 0）	2+2+2+0=6
Li H, et al. 2008 [34]	Untreated isolated systolic hypertension in elderly with SBP in the range of 140 to 179 mmHg	92.5	120	66.6	*Jiangya* capsule versus placebo	Nimodipine	4	BP; drug-related side effects	Baseline 24-hr SBPs: 152.6 vs. 153.5 mmHg; baseline 24-hr DBPs: 74.4 vs. 78.7 mmHg; follow-up SBPs: 134.2 vs. 141.9 mmHg; follow-up DBPs: 70.3 vs. 75.5 mmHg.	Positive	Yes (2/60 vs. 1/60)	2+2+2+1=7
Li H, et al. 2010 [35]	Untreated isolated systolic hypertension in elderly with SBP in the range of 140 to 179 mmHg	NA	270	66.9	*Jiangya* capsule versus placebo, nimodipine	None	4	BP; drug-related side effects	Baseline 24-hr SBPs: 142.2 vs. 143.2 vs. 145.6 mmHg (TCM + nimodipine simulation vs. nimodipine + TCM simulation vs. TCM + nimodipine); follow-up SBPs: 131.9 vs. 131.9 vs. 126.0 mmHg; SBP changes: -10.5 vs. -11.9 vs. -19.5 mmHg; DBP changes: -2.2 vs.-3.7 vs. -2.6 mmHg.	Positive	Yes (*Jiangya* capsules 2/90 vs. nimodipine 3/90)	2+2+2+1=7
Ma JP, et al. 2014 [36]	Untreated hypertension with BP in the range of 140/90 to 179/109 mmHg	80.0	118	61.4	*Qiqilian* capsule versus placebo	Nifedipine	4	Effective response; drug-related side effects	NA	Positive	Yes (1/54 vs. 0/64)	2+1+2+1=6
Chen X, et al. 2015 [37]	Untreated hypertension with BP in the range of 160/100 to 179/109 mmHg	29.3	199	46.7	*Jiangyabao* tablet versus placebo	Nifedipine; hydrochlorothiazide	24	BP; effective response; drug-related side effects	Baseline 24-hr SBPs: 141.5 vs. 138.8 mmHg; baseline 24-hr DBPs: 90.8 vs. 90.9 mmHg; follow-up SBPs: 128.1 vs. 129.7 mmHg; follow-up DBPs: 81.4 vs. 84.1 mmHg.	Positive	Yes (0 vs. 0)	2+2+2+1=7
Wang SH, et al. 2015 [38]	Untreated hypertension with BP in the range of 140/90 to 179/109 mmHg or BP higher than normal level with CCB or ACEI treatment	NA	240	NA (18-70)	*Tiankuijiangya* tablet versus placebo	CCB; ACEI	8	BP changes; effective response; drug-related side effects	SBP changes: -17.6 vs. -13.0 mmHg; DBP changes: -11.9 vs. -6.4 mmHg.	Positive	Yes (5/160 vs. 2/80)	2+2+2+1=7
Zhang DW, et al.	Essential hypertension (SBP of 140 to 159 mmHg, DBP	NA	338	54.8	*Angong jiangya* pill versus placebo	None	4	BP changes; symptom score;	SBP changes: -9.33 vs. -7.44 mmHg; DBP changes: -5.57 vs. -4.33 mmHg.	Positive	Yes (11/169 vs. 5/169)	2+1+2+1=6

2017 [39]	of 90 to 99 mmHg); Liver-fire Hyperactivity Syndrome (TCM)							Safety event evaluation				
Wang DH, et al. 2018 [41]	Essential hypertension (SBP of 140 to 159 mmHg, DBP of 90 to 99 mmHg)	NA	276	53.3	*Xuemaikang* capsule versus Losartan potassium	None	8	BP; symptom score	Baseline SBP: 145.70 vs. 145.12 mmHg; 8-week SBP: 134.91 vs. 135.11 mmHg; SBP Changes: -10.80 vs. -10.01 mmHg; Baseline DBP: 91.61 vs. 91.51 mmHg; 8-week DBP: 83.26 vs. 83.12 mmHg; DBP change: -8.36 vs. -8.39 mmHg.	Positive	Yes (0 vs. 0)	2+1+1+0=4
Zhang DY, et al. 2020 [40]	Masked hypertension, daytime ambulatory BP of 135 to 150 mmHg systolic or 85 to 95 mmHg diastolic and a clinic BP <140 mmHg systolic and 90 mmHg diastolic	NA	251	50.4	*Gastrodia-uncaria* granules versus Placebo	None	4	BP, Safety event	Baseline SBP: 135.6 vs. 134.4 mmHg; 4-week SBP: 130.2 vs. 131.5 mmHg; Changes: -5.44 vs. -2.91 mmHg; Baseline DBP: 89.8 vs. 89.1 mmHg; 4-week DBP: 86.4 vs. 87.5 mmHg; Changes: -3.39 vs. -1.60 mmHg.	Positive	Yes (0/126 vs. 1/125)	2+2+2+1=7
Lai XX, et al. 2022 [42]	Mild (grade 1) essential hypertension (SBP of 140 to 159 mmHg, DBP of 90 to 99 mmHg)	NA	628	52.5	*Songling xuemaikang* capsule versus Losartan	None	8	BP; Quality of Life and Sleep; symptom score; Serum Lipid Profiles	Baseline SBP: 145.2 vs. 144.1 mmHg; 8-week SBP: 134.7 vs. 133.5 mmHg; SBP changes: -10.5 vs. -10.6 mmHg; baseline DBP: 92.0 vs. 92.0 mmHg; 8-week DBP: 84.2 vs. 83.9 mmHg; DBP changes: -7.9 vs. -8.1 mmHg.	positive	Yes (6/314 vs. 8/314)	2+2+2+1=7
Macklin EA, et al. 2006 [43]	Untreated hypertension with BP in the range of 140/90 to 179/109 mmHg	NA	192	55.3	Active acupuncture versus Sham acupuncture	NA	52	BP; treatment-relatedserious adverse events	10-week SBP changes: -3.6 vs. -3.8 mmHg; 10-week DBP changes: -4.3 vs. -2.8 mmHg.	Negative	Yes (2/128 vs. 1/64)	2+2+2+1=7
Flachskampf, et al. 2007 [44]	Mild or moderate hypertension with BP in the range of 140/90 to 179/109 mmHg	NA	140	58.4	Active acupuncture versus sham treatment	NA	6	average SBP, DBP levels on 24-hour, average daytime and nighttime BP	Baseline SBP: 131 vs. 129 mmHg; 6-week SBP: 125 vs. 130 mmHg; 6-months SBP: 130 vs. 129 mmHg; Baseline DBP: 81 vs. 80 mmHg; 6-week DBP: 78 vs. 80 mmHg; 6-month DBP: 80 vs. 79 mmHg.	Positive (6week); Negative (after stop)	Yes（0 vs. 0）	2+2+2+1=7
Zheng H, et al. 2019 [24]	SBP from 140 to 159 mm Hg and/or DBP from 90 to 99 mm Hg	NA	428	59	Acupuncture group versus sham acupuncture, waiting-list group	NA	12	BP; Changes of visit to visit and 24hour BPV	6-week SBP changes: -7.2 vs. -4.1 vs. -4.1 mm Hg; 9-week SBP changes: -8.9 vs. -5.4 vs. -3.9 mm Hg; 6-week DBP: -3.7 vs.-0.8 vs. -3.3 mmHg; 9-week DBP: -4.3 vs.-1.8 vs. -3.9 mmHg.	Negative (6week); Positive (9week)	Yes (5/209 vs. 4/102)	2+2+2+1=7

ACEI, angiotensin converting enzyme inhibitors; BP, blood pressure; CCB, calcium channel blockers; DBP, diastolic blood pressure; NA, not available; SBP, systolic blood pressure; TCM, traditional Chinese medicine.

Supplementary Table S2: Characteristics of the randomized controlled trials of TCM for CHD

Studies included	Clinical diagnosis	Comorbidities	Patients (n)	Mean age (years)	Treatment comparison	Standard therapy	Follow-up (weeks)	Endpoints	Outcomes	Efficacy	Safety (Adverse events)	Modified Jadad score
Wang SH, et al. 2012 [58]	Stable angina	NA	66	61.5	*Danlou* tablet versus placebo	Antianginal drugs, aspirin, Simvastatin	4	Angina attack frequency, consumption of nitroglycerin, ST segment characteristics	Frequency: 7.0 vs. 16.5 times; consumption: 2.6 vs. 4.1 tablets.; ST segment: 2.6 vs. 3.2.	positive	NA	2+1+2+1=6
Zhang ZF, et al. 2015 [59]	Stable angina	NA	240	59.1	*Wufuxinnaoqing* capsule versus placebo	Antiplatelet drugs, ACEI/ARB, statins, and/or CCB	12	Angina attack frequency; duration of angina; consumption of nitroglycerin; cardiovascular adverse events	Total effective rate: 64.7% vs. 30.8%; angina symptom score: 44.9% vs. 25.4%; adverse events 7.56% vs. 7.50%; cardiovascular events: 0.84% (1/119) vs. 0 (0/120).	Positive	Yes (9/113 vs. 8/115)	2+2+2+1=7
Xu DP, et al. 2015 [60]	Stable angina undergoing PCI	Hypertension, dyslipidemia, diabetes, previous heart failure, arrhythmia, apoplexy, and/or gastroenteropathy	187	70	*Shenzhuguanxin* granule versus placebo	Antiplatelet drugs, nitroglycerin, statins, β-blockers, and/or CCB	52	Angina attack frequency; duration of angina; consumption of nitroglycerin; major cardiovascular events	Change APS scores: 2.41 vs. 3.14; emergency events of cardiovascular disease: 8.85% (10/113) vs. 16.22% (12/74).	Positive	Yes (10/113 vs. 12/74）	2+2+2+1=7
Gao J W, et al. 2018 [61]	Chronic stable angina	NA	232	NA	*Xinlin* pill versus placebo	Nitroglycerin	4	Total duration of treadmill exercise; consumption of nitroglycerin	Total exercise duration: 72. 11 vs. 31.25s; reduction of nitroglycerin: 2.45 vs. 0.50 tablets.	Positive	Yes (4/115 vs. 10/117)	2+2+1+1=6
Li Y, et al. 2019 [62]	Stable angina	MI, one major coronary branch stenosis ＞50%, PCI or coronary artery bypass grafting, typical angina symptoms	287	56	*Guanxinshutong* capsule versus placebo	Antianginal drugs, aspirin, β-blockers, CCB, statin drugs and ACEI/ARB.	4	Angina attack frequency at 4 weeks. consumption of nitroglycerin; score of SAQ; positive exercise tolerance text	Frequency per week: 9.4 vs. 2.5; SAQ score: 65.6 vs. 56.3; 1mm ST-depression: 219.7 vs. 237.7s.	Positive	Yes (7/143 vs. 3/144)	2+2+2+1=7

Zhao L, et al. 2019 [23]	Chronic stable angina for more than 3 months with attacks occurring at least twice weekly at baseline	NA	398	62.6	DAM group versus NAM group versus SA group versus WL group	Antianginal therapies β-blockers, aspirin, statins, ACEI	12	Frequency of angina attacks from baseline to 16 weeks; severity of angina as assessed with a visual analog scale	Baseline mean frequency: 13.31; frequency reduce: 7.96 vs. 3.89 vs. 2.78 vs. 2.33.	Positive	Yes (16/299 vs. 1/99）	2+2+2+1=7
Chu, et al. 2010 [63]	Unstable angina with blood stasis syndrome after PCI	NA	90	60.7	*Xuefuzhuyu* capsule, *Shengmai* capsule versus placebo	Injection of enoxaparin, aspirin, metoprolol tartrate, atrovastatin	4	Blood stasis syndrome scores. Short-form 36 and Seattle angina questionnaire score.	Ameliorating CSS and ECG were 76.7% and 60.0%, BSS scores: 21.3 vs. 28.3 vs. 33.7.	Positive	Yes (0 vs. 0 vs. 0)	2+2+2+1=7
Sun YL, et al. 2023 [64]	CHD, patients with angina pectoris twice or more per week angina grades are I-III, the angina grades are I-III	NA	324	63.3	*Suxiao Jiuxin* pill versus placebo	Conventional treatment for stable angina	24	Curative efficacy rate, total score of angina pectoris symptoms, SAQ score, TCM syndrome scores, adverse events	Curative efficacy rates: 21.57% vs. 30.07%; total score of angina pectoris symptoms: 3.64 vs. 4.80, SAQ score: 64.01 vs. 59.57, TCM syndrome scores: 2.47 vs. 2.90.	Positive	Yes (74/154 vs. 71/156)	2+2+2+1=7
Lu, et al. 2008 [69]	Previous MI	Hypertension and/or diabetes	4870	58.9	*Xuezhikang* capsule versus placebo	Aspirin, β-blockers, CCB, ACEI, and/or nitrates	234	Serum lipid levels and cardiovascular endpoints; drug-related side effects	Major coronary event：5.7% (138/2429) vs. 10.4% (254/2441), decreased CV and total mortality by 30% and 33%, LDL-C: -20% vs. -3.5%.	Positive	Yes (0 vs. 0)	2+2+2+1=7
Zhang, et al. 2010 [70]	STEMI undergoing primary PCI	Hypertension, hyperlipidemia, and/or diabetes	219	58.0	*Tongxinluo* capsule versus placebo	Aspirin, statins, clopidogrel, ACEI/ARB, β-blockers	24	Change of ST segment elevation; incidence of myocardial no-reflow.	Reduction of no reflow incidence: 34.26% vs. 54.05%; ST segment restoration in 6 hours: -0.22 vs. -0.18.	Positive	Yes (10/219)	2+1+2+1=6
Shang HC, et al. 2013 [72]	Previous MI	Hyperlipidemia, hypertension, diabetes, and/or gastritis	3505	58.3	*Qishenyiqi* dripping pill versus aspirin	Antiplatelet drugs, ACEI, CCB, β-blockers, diuretics, cardiotonic	78	Cardiovascular endpoints; drug-related side effects.	Incidences of the primary outcome 12 month: 2.98% (52/1746) vs. 2.96% (52/1759); 18-month: 3.67% (64/1746) vs. 3.81% (67/1759). Secondary	Positive	Yes (67/1746 vs. 95/1759)	2+2+2+1=7

						agents, nitrates, anti- arrhythmia agents, lipid lowering drugs			endpoints events 12-month: 2.41% vs. 2.44%; 18-month: 2.98% vs. 3.30%.			
Mao, et al. 2016 [73]	Acute myocardial infarction	NA	83	68	*Danlou* tablet versus placebo	NA	12	Left ventricular volumes, LV end-systolic volume index, LV ejection fraction	LVEDVi change: 4.49 vs. 0.34 mL/m²; volume index: -4.09 vs. -0.54 mL/m²; improved LV ejection fraction: 4.83 vs. 0.23%. Incidence of composite events: 11.90% (5/42) vs. 34.15% (14/41).	Positive	Yes (5/42 vs. 14/41)	2+2+2+1=7
Yang YJ, et al. 2023 [71]	Acute myocardial infarction	NA	3797	61.1	*Tongxinluo* versus placebo	STEMI guideline including dual antiplatelet therapy and coronary reperfusion	52	MACCEs at 30 days; individual components of the primary end point.	30 days MACCEs: 3.4% (64/1889) vs. 5.2% (99/1888); 1-y MACCEs: 5.3% (100/1889) vs. 8.3% (157/1888); 1-y all-cause death: 5.1% (97/1889) vs. 6.6% (124/1888).	Positive	Yes（Nonfatal serious adverse events: 2.2% vs. 2.8%, adverse drug reactions: 2.1% vs. 1.1%）	2+2+2+1=7
Lu XY, et al. 2006 [79]	CAD undergoing PCI	Hypertension, hyperlipidemia, diabetes, and/or previous MI	124	58.0	*Xiongshao* capsule versus placebo	Aspirin, heparin, ticlopidine, lipid lowering drugs clopidogrel, nitroglycerin,	26	Cardiovascular events; restenosis; drug related side effects	Incidence of RS: 24.1% vs. 48.5%, incidence of RS in stent: 14.0% vs. 42.0%; endpoint events: 11.7% vs. 27.6%.	Positive	Yes (0 vs. 0)	2+2+2+1=7
Li JG, et al. 2019 [80]	Stable coronary artery disease	Two or more of the conditions: a serum hs-CRP level ≥3 mg/L, hypertension, hyperlipidemia, and diabetes mellitus	1500	60.3	*Qingxin jieyu* granule versus placebo	Antiplatelet agents, RAAS lipid-lowering agents, blockers, antihypertensive agents, anti-diabetics.	52	Occurrence of composite events; including cardiovascular death, nonfatal MI; all-cause death.	Primary outcome: 0.67% vs. 1.33%; composite of all clinical outcomes: 1.9% vs. 3.68%; ‘hard’ endpoint was reduced by 0.99%.	Positive	Yes (12/750 vs. 23/750)	2+2+2+1=7
Wang DY, et al. 20210 [81]	Stable coronary artery disease	Two or more of the conditions: hs-CRP level >3 mg/L, hypertension,	114	61.6	*Yugengtongyu* granule versus Placebo	SCAD clinical guidelines	78	Cardiovascular death, any occurrence of all cause death	Incidence of composite outcomes: 5.26% (3/57) vs. 19.29% (11/57), major outcomes, independent events showed	Positive	Yes (4/57 vs. 7/57)	2+2+2+1=7

		hyperlipidemia, and diabetes mellitus							lowering trend.			
Wang, et al. 2023 [82]	CHD undergoing PCI	PHQ-9 scores between 5 to 14, or GAD-7 scores between 5 to 14	200	60.5	*Guanxindanshen* versus placebo	Followed AHA guidelines for CHD	12	PHQ-9 and GAD-7 scores; SAQ Score; MACEs; Safety event	PHQ-9 decreased: 3.97 vs. 1.18; GAD-7 score decreased: 3.48% vs. 1.13%; MACE: 1% (1/100) vs. 2% (2/100).	positive	Yes (24/100 vs. 19/100)	2+2+2+1=7
Shen ZJ, et al. 2020 [83]	Acute Coronary Syndrome with early PCI	Including acute STEMI, UA pectoris, or NSTEMI	200	61.2	*Suxiaojiuxin* pill versus placebo	Standard treatment of ACS	52	MACE, SAQ, safety assessment	Decreased the incidence of MACE; LVEF in 360 days: 60.99% vs. 58.83%; MACE (death, MI, stroke heart, failure rehospitalization): 1/2/12/5 vs. 6/4/9/14.	Positive	Yes (3/100 vs. 2/100)	2+2+2+1=7
Mehta, et al. 2014 [54]	Stable ischemic heart disease	Prior myocardial infarction, coronary angiography, angioplasty, or coronary bypass surgery.	151	63	TA versus sham acupuncture versus waiting control	SIHD clinical guidelines	12	24-Hour HRV, provocative testing HRV; exit resting heart rate, blood pressure, lipid profile, HOMA, hs-CRP, salivary cortisol, PAT, or psychosocial variables.	24-Hour HRV no differences, exit mental stress HRV was higher in TA vs. SA for markers of parasympathetic tone, including a 17% higher vagal activity.	Positive	NA	2+1+2+1=6
Ge JB, et al. 2021 [84]	Stable myocardial ischemia symptoms	Acute MI for > 6 months; PCI or CABG treatment > 6 months; stenosis of ≥50% in at least one major branch of coronary artery.	2674	63.8	*Shexiang baoxin* pill versus placebo	Standard treatment	104	MACE, all-cause mortality	MACE:1.9% (27/1335) vs. 2.6% (34/1327); occurrence of MACE reduced 26.9% after 2 years; all-cause mortality (0.37% vs. 0.23%), non-fatal MI (0.97% vs. 1.51%), and non-fatal stroke (0.67% vs. 0.90%).	Positive	Yes; least one AE: 236(17.7%) vs. 231(17.1%); SAE:47(3.5%) vs. 41(3.1%)	2+2+2+1=7
Zhou ZM, et al. 2023 [85]	Stable myocardial ischemia symptoms; history of DM or FBG ⩾ 7.0 mmol/L	Acute MI for > 6 months; PCI or CABG treatment > 6 months; stenosis of ≥ 50% in at least one major branch of coronary artery.	716	64.5	*Shexiang baoxin* Pill versus Placebo	Standard treatment	104	MACE, the composite outcome of all-cause death	MACE:2.6% (9/340) vs. 4.8% (18/376); risk of secondary outcome: 15.3% (52/340) vs. 22.6% (85/376)	Positive	Yes; least one AE 57(16.8%) vs. 18 (5.3%). Cardiovascular AE: 15/340 vs. 29/376	2+2+2+1=7

AHA, American Heart Association; ACEI, angiotensin converting enzyme inhibitors; ARB, angiotensin-receptor blockers; CABG, coronary artery bypass grafting; CCB, Calcium Channel Blockers; CHD, coronary heart disease; CM, Chinese medicine; DAM group, disease-affected meridian group; FBG, fasting blood glucose; GAD-7, generalized anxiety scale scores; HADS-a/HADS-d, hospital anxiety and depression scale; HRV, Heart rate variability; hs-CRP, high sensitive C reacting protein; MACCEs, major adverse cardiac and cerebrovascular events; MACE, Major Adverse Cardiovascular Events; NAM group, nonaffected meridian group; NSTEMI, Non-ST elevation myocardial infarction; PHQ-9, health questionnaire-9 scores; PCI, percutaneous coronary intervention; SAQ, Seattle Angina Questionnaire; SCAD, spontaneous coronary artery dissection; SIHD, stable ischemic heart disease; STEMI, Stable Angina to Myocardial Infarction.

Supplementary Table S3: Characteristics of the randomized controlled trials of TCM for stroke

Studies included	Clinical diagnosis	Comorbidities	Patients (n)	Mean age (years)	Treatment comparison	Standard therapy	Follow-up (weeks)	Endpoints	Outcomes	Efficacy	Safety (Adverse events)	Modified Jadad score
Han L. 2007 [89]	Ischemic stroke	Hypertension, diabetes, and/or CAD	180	NA (40-75)	*Naoxinduotai* capsule versus placebo	Nimodipine	4	Neurologic impairment score; Barthel index; drug-related side effects	Neurologic impairment score: 10.42 vs. 12.50, Barthel index: 70.18 vs. 65.43.	Positive	Yes (0 vs. 0)	2+1+1+1=5
Chen C, et al. 2009 [90]	Ischemic stroke	NA	605	59.1	*Danqi piantang* capsule versus placebo	NA	4	Neurological deficits, Motor Scores, Safety Results	Neurological deficits: 1.44 vs. 1.04.	Positive	Yes (2/400 vs. 0/205)	2+2+2+1=7
He L, et al. 2011 [91]	Ischemic stroke	NA	140	59.5	*Sanchitongshu* capsule versus placebo	Aspirin	24	European Stroke Scale; Barthel index; drug-related side effects	ESS changes: 19.85 vs. 12.07, BI changes: 26.34 vs. 20.94.	Positive	Yes (8/71 vs. 6/69)	2+2+2+1=7
Shen PF, et al. 2012 [25]	Ischemic stroke	NA	287	60.8	Acupuncture vs. Sham acupuncture	Defibrase, antiplatelet treatment	24	Recurrence and mortality, NIHSS, BI, SS-QOL	NIHSS in four weeks: 4.15 vs. 6.35, BI in six months: 70.25 vs. 57.43.	Positive	Yes (12/144 vs. 22/143)	2+2+2+1=7
Oskouei DS, et al. 2013 [92]	Ischemic stroke	Previous TIA, CAD, diabetes, hypertension, hyperlipidemia, previous intracranial hemorrhage, dysrhythmia	102	69.7	*Ginkgo* tablet versus placebo	NA	16	NIHSS	NIHSS scores changes: 4.7 vs. 4.1.	Positive	NA	2+2+1+1=6
Chen CL, et al. 2013 [93]	Ischemic stroke	Myocardial infarction, angina, hypertension	1099	61.4	*NeuroAiD* versus placebo	Standard stroke care, antiplatelet and control of vascular risk factors	12	mRS; NIHSS score; BI; safe events	No significance statistical in mRS, NIHSS and BI.	Negative	Yes (36/542 vs. 29/545)	2+2+2+1=7
CHIMES-E, 2015 [94]	Ischemic stroke	Previous hemorrhagic stroke, previous MI, angina, hypertension, diabetes,	880	61.8	*NeuroAiD* versus placebo	Antiplatelet drugs, control of vascular risk factors, and	104	Barthel index; mRS; death and occurrence of vascular events; drug-related side effects	Benefits of a 3-month treatment with MLC601 did not continue after 2 years. vascular event: 12.6% (56/446) vs. 12.7%	Positive	Yes (56/446 vs. 55/434)	2+2+2+1=7

		hyperlipidemia, and/or peripheral vascular disease				appropriate rehabilitation			(55/434); all-cause death: 6.3% (28/446) vs. 6.7% (29/434).			
Yu M, et al. 2015 [95]	Recent (<30 days) ischemic stroke in anterior cerebral circulation	Previous TIA, hypertension, diabetes, hyperlipidemia, previous MI, and/or angina pectoris	100	59.4	*Dihuangyinzi* tablet versus placebo	Rehabilitation and antiplatelet, lipid-lowering, antihypertensive, and antidiabetic medications	12	Fugl-Meyer assessment score; Barthel index; drug-related side effects	FMA score baseline: 48.0 vs. 51.0, 12week: 71.8 vs. 65.3, Barthel index score baseline: 44.1 vs. 46.2, 12-week score: 74.6 vs. 70.1.	Positive	Yes (6/45 vs. 5/42)	2+1+2+1=6
Wu Y, et al. 2021 [96]	Ischemic stroke	NA	288	59.1	*YangyinYiqi Huoxue* granule high/low dose vs. positive control vs. placebo	aspirin, dipyridamole, and ticlopidine	4	Comprehensive curative evaluation, NIHSS and CMS scores	Comprehensive curative effect evaluation:63.38% vs. 31.94 vs. 36.11 vs. 6.94; NIHSS score:4.49 vs. 5.71 vs. 5.97 vs. 6.97	Positive	Yes (0 vs. 0 vs. 3/72 vs. 1/72)	2+2+2+1=7
Wu LF, et al. 2023 [97]	Ischemic stroke	NA	2966	62	*Xuetaitong* granules + aspirin vs. Placebo+ aspirin	NA	12	Functional independence; all-cause death	Functional independence: 89% vs. 82%; all-cause death: 0.1% vs. 0.1%.	Positive	Yes (15/1488 vs. 16/1482)	2+2+2+1=7

CAD, Coronary Artery Disease; ESS, European Stroke Scale, FMA, Fugl-Meyer assessment; NIHSS, National Institute of Health stroke scale; SS-QOL, stroke-specific quality of life; TIA, transient ischemia attack

Supplementary Table S4. Characteristics of the randomized controlled trials of TCM for heart failure

Studies included	Clinical diagnosis	Comorbidities	Patients (n)	Mean age (years)	Treatment comparison	Standard therapy	Follow-up (weeks)	Endpoints	Outcomes	Efficacy	Safety (Adverse events)	Modified Jadad score
Zou X, et al. 2011 [105]	CHF with NYHA class of II to III	CAD and/or hypertension	150	69.7	*Nuanxin* capsule versus placebo	ACEI, β-blockers, diuretics, and/or digoxin	24	Effective response; NYHA functional classification; rehospitalization; acute heart failure; death; drug-related side effects	Quality of life scores: 22.18 vs. 28.25; rehospitalization: 23.9% vs. 53.4%; all-cause death: 5.63% vs. 20.54%.	Positive	Yes (1/71 vs. 0/73)	2+2+2+1=7
Wang C, et al. 2012 [106]	CHF with NYHA class of II to III	CAD	280	65.6	*Shencaotongmai* granule versus placebo	ACEI, diuretics, and/or digoxin	12	Effective response; NYHA functional classification; LVEF; drug-related side effects	LVEF increase: 6.55 vs. 3.14.	Positive	Yes (1/140 vs. 1/140)	2+2+2+1=7
Zhang Y, et al. 2012 [107]	CHF with NYHA class of II to III	NA	280	NA	*Qiangxintongmai* granule versus placebo	Lisinopril, hydrochlorothiazide and digoxin	12	Six-minute walk test; MLHFQ scores	Walk test: 412.75 m vs. 367.70 m; MLHFQ scores: 28.47 vs. 35.51.	Positive	NA	2+2+1+1=6
Li XL, et al. 2013 [102]	CHF with NYHA class of II to IV	AF and/or DM	512	57.3	*Qiliqiangxin* capsule versus lacebo	ACEI/ARB, β-blockers, aldosterone antagonists, diuretics, and/or digoxin	12	NYHA functional classification; LVEF; 6MWD; NT-proBNP; composite cardiac events; drug-related adverse events	NT-pro BNP: 240.15 pg/ml vs. 0 pg/ml; CCEs: 4.51% vs. 10.93%; SAEs: 4.8% vs. 9.4%; death: 2% vs. 3.6%.	Positive	Yes (19/250 vs. 23/250)	2+2+2+1=7
Fu XX, et al. 2014 [108]	CHF with NYHA class of II to IV	CAD, HHD1, RHD, PHD, and/or CM	140	64.0	Hot compress with *Zhuangshenling* formula versus placebo	ACEI, β-blockers, and/or diuretics	12	Effective response; NYHA functional classification; 6MWD; BNP	BNP after treatment: 156 pg/ml vs. 346 pg/ml; Walk test: 338.9 m vs. 323.3 m.	Positive	NA	2+2+1+1=6
Xian SX, et al. 2015 [110]	CHF with NYHA class of II to III	CAD, RHD, DCM, PHD, HHD1, and/or HHD2	228	68.3	*Yangxinkang* tablet versus placebo	Digoxin, ACEI/ARB, β-blockers, CCB, aspirin, diuretics, and/or spironolactone	4	Effective response; NYHA functional classification; physical examination; drug-related side effects	NYHA: 19.95 vs. 16.09.	Positive	Yes (0 vs. 0)	2+2+2+1=7

Wang X, et al. 2017 [109]	CHF with NYHA class of II to III;	Heart failure with frequent ventricular premature complexes	411	60.1	*Shensongyangxin* granule versus placebo	prescribed medications for CHF	12	Change of the total number of VPCs in24-h, LVEF, LVEDD, plasma NT-proBNP level, NYHA classification, 6MWD, MLHFQ scores	Total number of VPCs: 1,538 vs. 2,746; change of LVEF: 4.75 vs. 3.30; reduction in NT-proBNP: -122 pg/ml vs. -75 pg/ml; 6MWD: 35.1 m vs. 17.2 m.	Positive	Yes (39/210 vs. 52/201)	2+2+2+1=7
Mao, et al. 2020 [111]	Chronic ischemic heart failure	MI, one major coronary artery branch exceeding 50%, and coronary artery lesions likely closely correlated with HF	638	65	*QishenYiqi* dripping pills versus placebo	Standard international guideline directed medications for 2010	26	6-min walking distance, BNP, LVEF, NYHA and MLHFQ scores, composite endpoints	Walking distance change: 38.32 m vs. 6.31 m; 6 months composite endpoints: 13.17% vs. 16.61%; 6m cardiovascular events: 9.40% vs. 13.17%; death: 3.76% vs. 4.70%; 12m cardiovascular events: 15.67% vs. 19.12%; death: 5.33% vs 5.64%.	Positive	Yes (2/319 vs. 3/319)	2+2+2+1=7
Du KJ, et al. 2022 [112]	CHF	NA	191	70	*Qishen* granules versus placebo	Guidelines of CHF	12	Proportion of patients in NT-proBNP levels > 30%; NYHA, 6-min walking distance, MLHFQ scores	NT-proBNP: 891 pg/ml vs. 1,649 pg/ml; 6 MWD: 367 m vs. 333.5 m.	Positive	Yes (5/94 vs. 7/97)	2+2+2+1=7
Zhu MJ, et al. 2022 [113]	Ischemic heart failure	Myocardial infarction with or without PCI or coronary artery bypass grafting; ≥50% stenosis in at least one main coronary artery with or without revascularization	80	64.8	*Buyanghuanwu* versus placebo	Diuretics, ACEI or ARB, β-receptor blockers, aldosterone receptor antagonists, digoxin, and vasodilators	12	NYHA classification, TCM syndrome scores, NT-proBNP, 6MWD and LVEF	NT-ProBNP: 1,211.03 pg/ml vs. 1,931.23 pg/ml; 6 MWD: 435.91 m vs. 399.88 m; LVEF: 49.12 vs. 47.62.	Positive	Yes (NS)	2+2+2+1=7
Li XL, et al.2023 [114]	HFrEF, LVEF ≤ 40%; NT-pro BNP ≥ 450 pg/ml	NA	3110	62	*Qiliqiangxin* capsule versus placebo	Standardized treatment of chronic HF	36 m	MACE, all-cause death; cardiovascular death, second composite points, NT-proBNP	MACE: 25.02% vs. 30.03%; All-cause death: 14.21% vs. 16.85%; NT-pro BNP: -444 pg/ml vs. -363 pg/ml.	Positive	Yes (NS)	2+2+2+1=7

ACEI, angiotensin converting enzyme inhibitors; AF, atrial fibrillation; ARB, angiotensin-receptor blockers; BNP, brain natriuretic peptide; CAD, coronary artery disease; CCB,

calcium channel blockers; CCEs, composite cardiac events; CHF, chronic heart failure; CM, cardiomyopathy; DCM, dilated cardiomyopathy; DM, diabetes mellitus; HF, heart failure; HHD1, hypertensive heart disease; HHD2, hyperthyroid heart disease; LHFQ, Living with heart failure questionnaire; LVEF, left ventricular ejection fraction; 6MWD, 6-min walking distance; MLHF, Minnesota Living with Heart Failure; NA, not available; NS, Not significant; NT-pro BNP, N-terminal prohormone of brain natriuretic peptide; NYHA, New York Heart Association; PHD, pulmonary heart disease; RHD, rheumatic heart disease; SAEs, serious adverse events; TCM, traditional Chinese medicine; VHD, valvular heart disease.
